# Longitudinal Substance Use and Biopsychosocial Outcomes Following Therapeutic Community Treatment for Substance Dependence

**DOI:** 10.3390/jcm9010118

**Published:** 2020-01-01

**Authors:** Petra K. Staiger, Paul Liknaitzky, Amelia J. Lake, Stefan Gruenert

**Affiliations:** 1 School of Psychology, Deakin University, Geelong 3220, Australia; paul.liknaitzky@deakin.edu.au (P.L.); alake@acbrd.org.au (A.J.L.); 2 Deakin University Centre for Drug Use, Addictive and Antisocial Behaviour Research (CEDAAR), Burwood 3125, Australia; 3 Odyssey House Victoria, Melbourne 3121, Australia; sgruenert@odyssey.org.au; 4 The Australian Centre for Behavioural Research in Diabetes, Diabetes Victoria, Melbourne, Victoria 3051, Australia

**Keywords:** Therapeutic Community, substance use disorder, residential rehabilitation, outcomes, drug and alcohol

## Abstract

The Therapeutic Community (TC) model is considered an effective treatment for substance dependence, particularly for individuals with complex presentations. While a popular approach for this cohort across a number of countries, few studies have focussed on biopsychosocial and longer-term outcomes for this treatment modality. This study reports on substance use, dependence, and biopsychosocial outcomes up to 9 months post-exit from two TC sites. Methods: A longitudinal cohort study (*n* = 166) with two follow-up time points. Measures included substance use, dependence, subjective well-being, social functioning, and mental and physical health. Generalized Linear Models were employed to assess change over time. Results: At 9 months, 68% of participants reported complete 90-day drug abstinence. Alcohol frequency and quantity were reduced by over 50% at 9 months, with 32% of the sample recording 90-day abstinence at 9 months. Both alcohol and drug dependence scores were reduced by over 60%, and small to medium effect sizes were found for a range of psychosocial outcomes at 9 months follow-up, including a doubling of wellbeing scores, and a halving of psychiatric severity scores. Residents who remained in the TC for at least 9 months reported substantially better outcomes. Conclusions: With notably high study follow-up rates (over 90% at 9 months post-exit), these data demonstrate the value of the TC model in achieving substantial and sustained improvements in substance use and psychosocial outcomes for a cohort with severe substance dependence and complex presentations. Implications for optimal length of stay are discussed.

## 1. Introduction

Originally designed to be implemented in a psychiatric setting, the concept of the Therapeutic Community (TC) has evolved to be applied to drug rehabilitation [1]. Central to the TC approach is the view that substance dependence relates to fundamental issues within an individual’s lifestyle and self-identity, more so than to the addictive profile of particular drugs [2]. Consequently, a key characteristic of TC treatment is its focus on lifestyle and identity change using the ‘community as method’, a treatment environment that seeks to ameliorate psychological problems, enhance social functioning, facilitate behavioural changes, and ultimately lead to a drug-free lifestyle. The TC approach is a highly structured and primarily self-governed community, which distinguishes it from other types of residential rehabilitation, with clear expectations, consequences, roles, and schedules, in which residents progress through a hierarchy of increasing responsibilities. These structures help residents integrate into social networks, increase social skills, enhance accountability to the group, and instil self-reliance [3]. This study sought to determine substance use and biopsychosocial outcomes at 3 months and 9 months following TC treatment across two sites. Furthermore, the length of planned TC treatment generally varies between four weeks and 12 months [4,5,6]. This study sought to determine the role of length of stay in clinical outcomes.

TC effectiveness is typically assessed by rates of abstinence from drugs or alcohol, or various measures that indicate a substantial reduction (e.g., 50%) in use and related harm. However, there is an increasing focus on reporting psychosocial outcomes, consistent with TC philosophy [7,8,9], although most reviews tend to focus on substance use outcomes only. Systematic reviews of TC outcomes have generally found support for their effectiveness across a wide range of populations and contexts [10,11]. One notable exception is a Cochrane Review by Smith et al. [12] that found that TCs tended to show limited success compared to other residential formats. However, this review has been criticised [10,11] due to their broad inclusion criteria (i.e., including prison-based TCs), which limits the conclusions that can be drawn in relation to voluntary, community-based TCs. Malivert and colleagues [10] addressed these concerns within a systematic review by excluding prison-based TCs and including any TC study with post-discharge outcome measures. Across 12 studies, they found significant reductions in drug use post-discharge, but overall these reductions were not always greater than those reported in other treatment modalities. However, they point out that the unique benefit of TCs is that they may provide the best treatment option for those with severe substance dependence and complex psychosocial presentations. Interestingly, a recent large-scale substance use treatment study from Australia found that abstinence from alcohol was more likely to be reported if an individual’s treatment history included a residential stay (in Australia, these are primarily TCs) [13]. Finally, although the most recent systematic review of TCs for substance use disorders reported variable outcomes across studies [11], the authors concluded that TCs were generally effective for reducing substance use and criminality, and in some studies (when measured) improving mental health outcomes and social functioning. Overall, the literature suggests that TCs are an effective treatment option with some indication that this is particularly the case for those with severe substance use and complex psychosocial presentations.

Length of stay (LOS) in TCs has been linked to improvements in substance use, criminality, employment, and other psychosocial outcomes [14,15,16,17], even after controlling for other key predictors [9,14,18,19]. For example, for each additional month within a TC, at 1-year follow-up the odds of any heroin use decreased by 6% [14]. To improve outcomes, early studies suggested various minimum TC treatment durations, ranging from 50 to 365 days [5,20,21]. With limited empirical basis, three months became a common heuristic for minimum LOS [9,14,19,22], although no clear consensus has been established [17]. While further controlled trials are warranted, similar links between LOS and improvements in psychosocial outcomes have been reported [9,23].

Surprisingly, although TCs have been widely implemented across a range of countries, there is a dearth of outcome studies that report post-exit follow-up data greater than six months. One recent multi-site Australian study found that residential rehabilitation (including TCs) was associated with higher odds of abstinence at 12 months follow-up than acute withdrawal services for drugs and alcohol, and higher odds of abstinence than acute withdrawal and outpatient services when alcohol was the primary drug of concern [13]. However, this study did not distinguish TCs from other types of residential rehabilitation. Another study showed clinically significant improvements in psychosocial and wellbeing outcomes on exit from a TC [7]. However, this paper did not report substance use outcomes, or any follow-up measures.

The current study goes some way towards addressing the current gap in the literature by reporting on: TC outcomes specifically (not combined with other residential treatments), substance use and biopsychosocial outcomes, magnitude and clinical significance of outcomes, and mid- to long-term follow-up data. Specifically, this paper reports on the 3 months and 9 months TC outcome data from a twin-site study conducted in Australia. The primary outcomes examined were: drug and alcohol use; and drug and alcohol dependence. Secondary, biopsychosocial outcomes were: mental health; subjective wellbeing; physical health; social functioning; and employment.

## 2. Method

### 2.1. Participants

Residents who were undergoing residential treatment for substance dependence within a TC facility (Table 1) were invited to participate. Inclusion criteria were almost identical to those of TC eligibility criteria to ensure findings had broad relevance: being 18-years and over, spoken English, and fulfilling Diagnostic and Statistical Manual of Mental Disorders (DSM-IV) criteria for substance dependence disorder in the previous 12 months [24]. The exception was that those with major neurological issues, psychosis symptoms, or suicidality were excluded based on clinical assessment of the admitting team. Participants were compensated for their time with an AUD$20 voucher at 3 months follow-up and an AUD$30 voucher at 9 months follow-up.

### 2.2. Procedure

Ethics approval was obtained from the relevant university committee (DUHREC_EC20047) and written informed consent was received from all participants. All participants completed a self-report survey and participated in a face-to-face interview 3–4 weeks post-TC admission (to allow for stabilisation and adjustment). Baseline drug/alcohol use was reported for the 3-month period immediately prior to entering the TC (excluding any incarceration/detoxification period). Interviews were conducted by one of four researchers with post-graduate qualifications in psychology.

There were two follow-up assessments: 3 months and 9 months post-exiting the TC. Follow-up interviews were conducted face-to-face; however, where the person was unwilling/unable to attend, interviews were conducted over the phone. This study draws on the data from a funded project examining the impact of a brief alcohol intervention, the findings of which are reported elsewhere [25].

### 2.3. Therapeutic Community Treatment Program

Therapeutic communities are a sub-set of drug-free, residential drug treatment programs. They are highly supervised and structured environments which combine medical, psychological and peer support, with meaningful work activities and therapeutic groups. Initially, residents have minimal contact with external networks as they develop peer relationships and life skills, a positive daily routine and work ethic, and an understanding of their own individual needs. Utilising ‘the community’ as the method or agent of change, TCs comprise a number of stages that individuals work through, leading to increasing self-responsibility, privileges, and responsibility to others. Compared to other types of residential rehabilitation programs, residents in a TC are given greater responsibility for the management and operation of the community, and for providing mutual support and feedback. They contribute to all aspects of the functioning of the community including cooking, cleaning, support, teaching, and maintenance. Consistent with the social identity model of recovery [26], the underlying philosophy is that drug use occurs as a result of a disruption to healthy social and emotional development. Most of the residents have a history of trauma, abuse and neglect, and often lack the self-regulation skills required to develop and maintain positive relationships or function in the community without the use of drugs or alcohol. The two TCs participating in this study are considered modified TCs that have both been certified under the Australasian Therapeutic Communities Association Standard. They are set on large, semi-rural properties with animals and vegetable gardens, and they incorporate psycho-social skills training and some aftercare support. Their residents stay for anywhere between three to 15 months before being supported to transition into housing, training and/or employment. Both TCs employ a significant number of staff members with a lived experience of addiction (and a minimum of one year in recovery), including program graduates who undergo alcohol and other drug studies.

### 2.4. Measures

Demographic data were collected at baseline including age, gender, ethnicity/nationality, country of birth, marital status, level of education achieved, occupation, employment status, welfare benefits, living conditions, primary substance of abuse, age at onset of substance abuse problems.

Timeline Follow-Back (TLFB): The TLFB method [27] was used as a measure of frequency of drug use, and quantity and frequency of alcohol use over the preceding 90-day period. The TLFB is well validated and has been used extensively [28]. We computed ‘drug episodes’ as a more accurate measure of drug use that takes into account polydrug use. In this respect, ‘drug episodes’ over the three-month period was defined as the sum of the number of days of use for each drug type (excluding alcohol). For example, three drug episodes could refer to a single drug type used on three separate days, or alternatively to three separate drug types used on a single day. Similarly, an ‘alcohol episode’ referred to a day when alcohol was used. Alcohol was reported separately to drugs in order to represent both the frequency and quantity of alcohol consumption.

Severity of Dependence Scale (SDS): The SDS [29] has five items measuring level of dependence, with a focus on impaired control of drug use. It has been used in samples with a variety of drug dependencies and has been found to have good psychometric properties, with high internal consistency across items (α = 0.8 to 0.9), and good construct validity [29]. Cut-offs of 3 to 5 on the SDS are usually indicative of clinical dependence, depending on the drug [30,31,32].

Severity of Alcohol Dependence Questionnaire (SADQ): The SADQ [33] is a 20-item questionnaire measuring severity of alcohol dependence. It has good psychometrics, with an 82% concordance between clinician’s ratings of alcohol dependence and SADQ scores [34]. Scores of 31 and above are indicative of severe alcohol dependence [33].

Opiate Treatment Index (OTI)—social functioning subscale: The OTI [35] is a self-report multi-dimensional instrument with excellent psychometrics [36]. This trial utilised the 12-item social functioning subscale, which assesses residential stability, employment, inter-personal conflict, social support, and drug culture involvement. The social functioning subscale has excellent test–retest reliability (ranging from 0.85 to 0.89); correlates significantly with the Addiction Severity Index (0.42), supporting validity; and internal reliability was found to be moderate (α = 0.58; [37]).

Personal Wellbeing Index (PWI): The PWI [38] is an 8-item questionnaire probing satisfaction, with eight quality-of-life domains: standard of living, health, life achievement, personal relationships, personal safety, community-connectedness, future security and spirituality-religion. The PWI has been used extensively across cultures [39] and has good psychometric properties [38]. The scale has high internal consistency, with Cronbach’s alphas between 0.70 and 0.85; and it correlates 0.78 with the Satisfaction with Life scale, demonstrating convergent validity [38].

Addiction Severity Index (ASI): The ASI [40] is a structured clinical interview designed to evaluate treatment outcomes for substance-dependent individuals. The ASI has been used extensively in both clinical practice and research on substance use, including dual diagnosis. This study used the medical (hospitalisations, medication, disability support, frequency and severity of problem), employment status (days of employment, government benefits, income), and psychiatric symptoms subscales (psychiatric symptoms, violence, suicidality, medication, frequency and severity of symptoms). The ASI has good test–retest reliability and excellent concurrent reliability with an average concordance of 0.89 with trained clinicians assessing severity of addiction problems [41].

### 2.5. Data Analytic Procedure

Data were analysed using R (R Foundation for Statistical Computing, Vienna, Austria) [42] and SPSS version 25 (IBM Corp, Armonk, NY, United States). Missing data were found to be missing at random, and were replaced using the Predictive Mean Matching (PMM) method for Multiple Imputation [43]. PMM draws on real values in the dataset, thereby avoiding issues with negative or fractional count of drug episodes. Consumption use variables were mostly count variables (number of drug episodes) with over-dispersion and zero inflation at follow-up data points. Accordingly, we used negative binomial Generalized Linear Models (GLM) with log link functions to model change over time. These models were replaced by Tweedie models for several non-count variables that conformed to Tweedie distributions. Where multiple post-hoc comparisons were made, the False Discovery Rate method [44] was used, which corrects for the expected proportion of Type-1 errors. Cohen’s *d* effect sizes were calculated for 9 months post-TC exit.

## 3. Results

### 3.1. Baseline Descriptive Statistics

Of the 166 eligible residents who provided baseline data over a 23-month period (2007–2009), 95% (*n* = 157) and 90% (*n* = 150) provided 3- and 9-month data respectively. The majority of 3 months and 9 months interviews were conducted in person (82% and 84%, respectively). Main reasons for telephone interviews were: no fixed address, incarcerated or living outside metropolitan area.

Baseline demographic and substance use data are presented in Table 1. The mean number of drug episodes (not including alcohol) was 8.0 per week, the mean number of alcohol episodes was 3.6 per week, with a mean of 14 Standard Drinks consumed per drinking day (Table 2). For the subsample who identified as having a primary alcohol issue with or without comorbid drug dependence (*n* = 30), the mean number of drinking days was 5.5 per week, with a mean of 25 Standard Drinks consumed per drinking day. The most frequently consumed substances in the 90 days prior to TC entry were alcohol (mean proportion of days = 0.51), cannabis (mean proportion of days = 0.35), heroin (mean proportion of days = 0.24), and amphetamine (mean proportion of days = 0.21). Level of alcohol and other drug dependence was very high in this cohort; for example, the group mean SDS score was higher than scores typically reported in drug treatment-seeking individuals, with 75.3% of all participants scoring 5 and above at treatment entry (i.e., high levels of drug dependence). For those individuals with a primary alcohol problem, 77% of this subsample (*n* = 30) were considered severely alcohol dependent (scores above 30 on the SADQ). Baseline wellbeing in this cohort was markedly low, with only 7.2% of residents scoring within the range of normative Australian individual scores (50.4 to 100, across 30 studies, *n* = 59,536; [45]), and with a substantially lower group mean PWI score (mean = 25.2) than found in other substance misuse cohorts (means from 47.9 to 55.4; [46,47,48]) (see Table 3).

### 3.2. Primary Drug and Alcohol Outcomes: Use and Dependence

Using GLM, we assessed change in alcohol and drug consumption, and level of dependence at both 3 months and 9 months post TC exit (see Table 2 for statistical outcomes). The mean number of drug episodes (excluding alcohol) was significantly reduced (by 77% from baseline to 3 months, sustained at 9 months). In terms of clinical significance, 90-day drug abstinence at 3 months and 9 months was achieved by 49% and 57% of the sample, respectively. Of note, when considering only participants with drug use at baseline (*n* = 142), 58% were completely abstinent at 3 months and 68% at 9 months, also a large effect size. Further, drug consumption was reduced to over half of baseline levels at 3 months and 9 months in 77% and 82% of the sample, respectively. For the subsample who were not abstinent at follow-up, mean drug episodes dropped by 62% at 3 months and by 52% at 9 months. No significant differences were found for abstinence rates or 50% reduction rates between 3 months and 9 months, indicating that sustained change was achieved by participants.

In terms of alcohol consumption, the number of drinking days was significantly reduced by 60% from baseline to 3 months, and by 57% from baseline to 9 months (Table 2). At both follow-up time points, the mean number of drinks per drinking day decreased significantly to 49% of baseline levels. For the subsample (*n* = 30) who identified as having a primary alcohol issue (includes four individuals with comorbid drug dependence) alcohol frequency was reduced by 70% from baseline to 3 months and 9 months (5.5 drinking days per week at baseline to 1.7 at 3 months and 1.6 at 9 months), with alcohol quantity reduced by 60% from baseline to 3 months and 9 months (25.2 Standard Drinks per drinking day at baseline to 10.2 at 3 months and 10.6 at 9 months). In those with a primary alcohol problem, abstinence rates were 37% at 3 months and 23% at 9 months. In general, the post-treatment alcohol goal for those dependent on drugs was reduction in consumption, rather than abstinence. In this context, 32% of the total sample were completely abstinent from alcohol for the previous 90 days at both 3 months and 9 months. Overall, alcohol consumption was reduced by at least 50% in 63% of the total sample at 3 months and 64% of the sample at 9 months.

**Figure 1:**
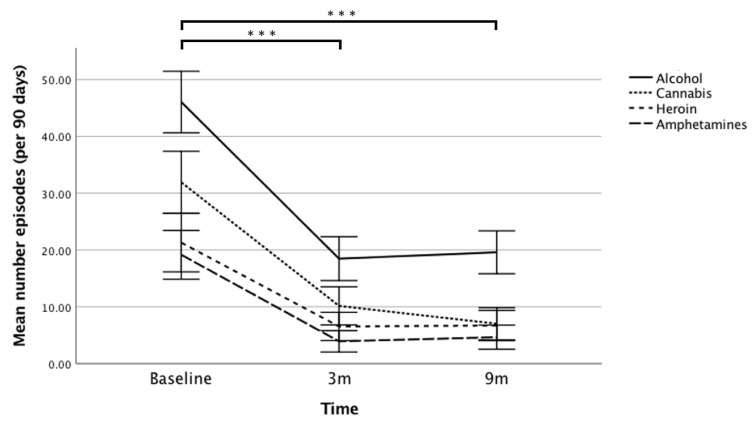
Mean number of episodes, separated by substance type (alcohol, cannabis, heroin, and amphetamine), at baseline, 3 months (3m) follow-up, and 9 months (9m) follow-up. Error bars = 95% Confidence Interval (CI) of mean, *** *p* < 0.001 for each of the four drug types, See Table S1 in Supplementary Materials for detailed statistics.

Mean drug dependence was significantly reduced by 55% from baseline to 3 months, which improved further to a 64% reduction at 9 months. Similarly, mean alcohol dependence was significantly reduced (by 61%) from baseline to 3 months, and maintained at 63% of baseline at 9 months. The percentage of participants scoring above the more conservative clinical cut-off of 5 on the SDS was 73% at baseline, dropping to 36% at 3 months, and 29% at 9 months. For the SADQ, 29% of the sample scored above the cut-off of 31 for severe alcohol dependence at baseline, dropping to 3% at 3 months, and 2% at 9 months. In terms of the sub-sample with a primary alcohol or both a primary alcohol and drug issue, the percentage above this cut-off was 77% at baseline, dropping to 3% at both 3 months and 9 months. Finally, separating consumption episodes by drug type, medium to large reductions across the sample were recorded at 9 months for cannabis (*d* = −0.83), amphetamines (*d* = −0.79), heroin (*d* = −0.63), and alcohol (*d* = −0.47; Figure 1).

### 3.3. Secondary Outcomes: Biopsychosocial Variables at 3 Months and 9 Months Follow-Up

Using GLM, we examined changes in biopsychosocial outcomes at 3 months and 9 months (Table 3). Models showed statistically significant improvements in four (psychiatric status, employment status, social functioning, wellbeing) out of five biopsychosocial outcomes at both 3 months and 9 months. By way of clinical illustration, a number of distinct survey items from the ASI are described. For example, the number of days reported experiencing psychological and emotional problems over the past 30 days was significantly reduced from 22.3 (SD = 10.9) at baseline to 13.2 (SD = 12.2) at 3 months to 11.0 (SD = 11.7) at 9 months. To a lesser degree, small improvements in employment status were reported; mean number of days in paid work over 30 days increased from 5.0 (SD = 9.3) at baseline to 7.2 (SD = 9.4) at 3 months and 8.7 (SD = 10.2) at 9 months. No significant differences from baseline were found for medical status at 3 months and 9 months. Social Functioning as measured by the OTI subscale improved significantly by 25% at 3 months and by 34% at 9 months respectively. Finally, mean Wellbeing as measured by the PWI more than doubled at 3 months and 9 months.

### 3.4. The Effect of Length of Stay (LOS) on Substance Use Outcomes

**Figure 2:**
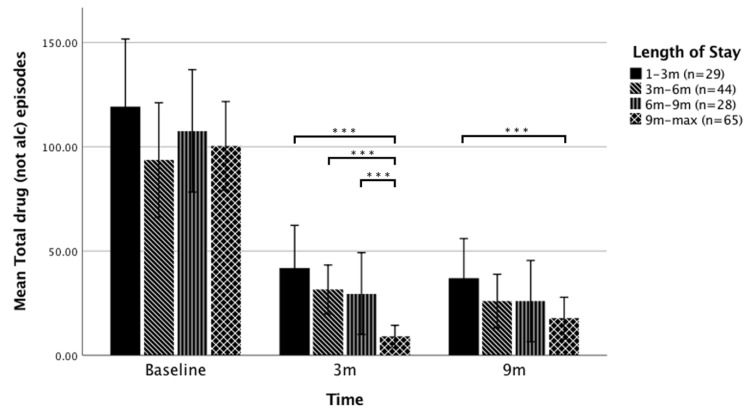
Mean number of drug episodes over the previous 90 days as a function of treatment duration at baseline, 3 months (3m) follow-up, and 9 months (9m) follow-up. Note, Error bars = 95% Confidence Interval of mean, *** *p* < 0.001, See Table S2 in Supplementary files for detailed statistics.

The median LOS for study participants was 206.5 days (IQR = 104.8–370.0), or approximately 9 months. Participant LOS was grouped as follows: 89 days or less (1–3 months; *n* = 29), 90 to 179 days (3–6 months; *n* = 44), 180 to 269 days (6–9 months; *n* = 28), and 270 days or more (9+ months; *n* = 65). Using a negative binomial GLM and correcting for multiple comparisons using the False Discovery Rate method, significant LOS group differences were found for number of drug episodes at 3 months and 9 months. Participants who remained at the TC for longer than 9 months showed a 74% greater reduction in mean drug episodes at 3 months than the mean of the other treatment duration groups combined. At 9 months follow-up, this difference was still significant when comparing participants who remained at the TC for longer than 9 months compared with those who remained for shorter than 3 months, with a 52% greater reduction in drug episodes for the +9 months group compared with the under 3 months group (Figure 2).

**Figure 3:**
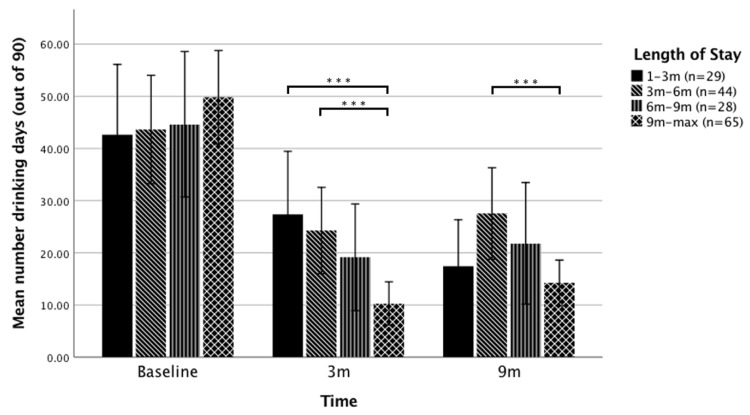
Mean number of drinking days over the past 90 days as a function of treatment duration at baseline, 3-month follow-up, and 9-month follow-up. Note, Error bars = 95% Confidence Interval (CI) of mean, *** *p* < 0.001, 3m: 3 months, 9m: 9 months. see Table S2 in Supplementary Files for detailed statistics.

For alcohol consumption, significant LOS group differences were found for number of drinking days at 3 months and 9 months. Specifically, compared with those who remained within the TC for under 3 months and 3–6 months, participants residing at the TC for over 9 months reported 60% fewer drinking days at 3 months (Figure 3). At 9 months, a significant 48% reduction in drinking days was found for participants staying over 9 months compared with those who stayed 3–6 months. No significant LOS group differences were found for the average number of Standard Drinks consumed per drinking day, for drug dependence (SDS), or for alcohol dependence (SADQ), after correcting for multiple comparisons.

## 4. Discussion

The key findings in this large TC outcome study were substantial and meaningful reductions in drug and alcohol consumption, dependence scores, and a range of biopsychosocial outcomes at 3 months post-TC exit, sustained at 9 months. Secondly, consistent with other residential treatment studies [9,14,15,16,23], length of stay (LOS) predicted improved outcomes, with the data generally showing a linear ‘dose–response’ relationship, with maximal gains for individuals who stayed in the TC for over 9 months. Furthermore, this cohort presented with substantial socio-economic disadvantages and markedly low personal wellbeing compared to most other treatment seekers in community-based alcohol and drug services (see below for details). For example, the majority of residents were not in a relationship, were unemployed, and had not completed high school. It is important that the outcomes reported in this paper are considered in the context of this substantially underprivileged cohort of individuals, exhibiting moderate to severe levels of drug dependencies, polydrug use, and psychological comorbidities.

Despite the severity and presentation complexity in this population, 68% of TC residents who used drugs at baseline reported 90-day abstinence at 9 months post-exit. Across the whole sample, alcohol use frequency was reduced by 57% and alcohol quantity by 49%. For residents with a primary alcohol issue, alcohol frequency was reduced by 70% and alcohol quantity by 60%. Significant medium to large effect sizes were found for baseline to 9-month consumption changes for all measured drug types: alcohol, heroin, amphetamines, and cannabis. The largest effect size was reported for those whose primary drug of choice was amphetamines, with the lowest for alcohol (a medium effect size; see Supplementary Materials). Importantly, these effect sizes are larger than what is generally achieved in non-residential settings.

Of the five biopsychosocial measures, four showed significant improvements at both follow-up time points. Wellbeing (PWI) improved by 112% at 9 months post-exit from the TC. Wellbeing scores at baseline in this cohort (mean PWI = 25.2) were markedly lower than normative scores across 30 general population studies in Australia (mean PWI ~ 75; *n* = 59,536; [45]), and about half of the typical baseline scores seen in treatment-seeking substance dependent individuals (mean PWI ~ 50) [46,47,48]. Social Function scores improved by 34% at 9 months post TC. Baseline scores on this subscale (mean OTI = 20) were comparable to similar psychosocially complex substance-dependent groups (34). Psychiatric symptoms as measured by the ASI improved by 44% from baseline at 9 months post-exit, comparable to other TC studies [8,49]. However, Psychiatric Status at baseline in this cohort (mean = 0.6) was substantially worse than a range of different substance dependent cohorts in both TC and non-TC service settings: M = 0.3 in (45); M = 0.4 in (8); M = 0.2 in [48]. Employment Status improved by 19%, similar to those following residential treatment in a recent TC study [8]. Although promising, this outcome is low compared with substantial improvements in the other outcome variables, and suggests that re-entering the employment market following lengthy residential treatment may be particularly challenging, and requires additional targeted support. For example, tailored and government-supported programs have been shown to assist prisoners with employment reintegration while reducing recidivism [50]. Medical Status at baseline, similar to previous pooled data across substance dependence treatment programs [51], did not improve following treatment. As many TC residents have chronic or recurring medical issues (such as Hepatitis-C and liver disease), their improvement may require a longer period of time, or specific and specialised treatments that were not provided by the TCs at the time of this study, (current Hepatitis-C treatment was not available at the time).

A key finding here was that increased LOS was associated with generally better outcomes across a range of domains. This has been shown in a number of previous TC studies, and even while controlling for other predictors [9,15,16,18,19], a link that may be more important in TCs than in some other long-term residential programs [14]. In contrast to the common recommendation of a 90-day treatment program within TCs (e.g., [22]), the results from this study suggest increasing improvements in substance use over time with 9 months or longer of treatment, in line with another recent TC outcome study [9]. Moreover, while some researchers have suggested that significant results can only be achieved within a TC following a minimum LOS of 90 days (e.g., [22]), our results indicate that reductions in substance use can be found in residents who stayed for under 90 days, but these improvements are only half of what is achieved compared to those staying longer than 9 months. It is interesting to note that TC outcomes studies that do not analyse outcome differences as a function of LOS, may obscure some of the more positive findings associated with individuals who stay for longer periods.

It is critical when examining TC outcome research to understand that people accessing TC treatment tend to have more complex psychosocial concerns than people attending outpatient treatments and other shorter-term residential treatment [52]. The TC approach may be particularly well-adapted to address more complex and socio-economically disadvantaged client groups. For example, one study found that clients with polydrug problems, medical issues, and legal problems were more likely to have significantly better outcomes in TCs compared with Twelve-Step and other models of treatment [53]. This study was no exception, whereby residents at baseline reported excessively high levels of drug and/or alcohol use and high levels of dependence, almost all were polydrug users and on government benefits, with very few fully employed. Further, baseline biopsychosocial scores were all low, with extremely low levels of wellbeing even compared with other residential treatment cohorts. While not suggesting that TCs are only suited for individuals at the severe and complex end of the spectrum, this study shows that TCs are able to assist these individuals to make substantial and sustained changes across a range of critical aspects of their lives.

### 4.1. Study Design Strengths and Limitations

Noteworthy strengths of this study are the high rates of baseline recruitment (90% of eligible residents) and follow-up (95% and 90% at 3 months and 9 months, respectively), which are higher than similar studies of complex polydrug users [13,54]. Substantial effort by investigators to follow up with participants contributed to the low attrition rates. Consequently, the findings reported can be viewed with confidence and are likely to generalise to the TCs within Australia and beyond. Importantly, the study provides strong evidence of very positive and sustained outcomes, across two TCs, supporting a diverse application of the TC model. The trial also sought to determine various biopsychosocial outcomes, extending beyond the commonly investigated substance use and dependence indicators. While a positive association between LOS and clinical improvements has been found in previous studies [17], results have often been limited by potential response bias due to high attrition rates [6,9]. The very low attrition rates in this study add more reliable support to the link between increased LOS and clinical improvements.

One potential limitation was that intake into this study occurred after residents had been at the Therapeutic Community for about 3–4 weeks. Consequently, these data represent a sample who have had a ‘minimum dose’ of at least 3-weeks in the TC. Also, baseline reporting for the period prior to entry to inpatient treatment programs can be affected by both escalated consumption (prior to planned abstinence) or decreased consumption (through preceding detox and other programs). While this may impact data in this study, we addressed this in part by measuring baseline data for the period prior to any detoxification that immediately preceded entry into the TC. Furthermore, due to a lack of control group, it is not possible to separate out the effects of the TC from spontaneous recovery, however, the strong dose–response effect reported in this study indicates that a substantial proportion of the outcomes is likely to be attributable to treatment, rather than to natural recovery. Additionally, previous work has shown that changes in one’s sense of identity (away from a ‘user identity’ and towards a ‘recovery identity’) predict commitment to sobriety and improved wellbeing within TCs [55]. In line with social identity frameworks, changes to an individual’s identity may in part depend on changes to the groups identified with. The shift away from identifying with a group of known drug users to a new group of people in a residential treatment context can take time. It is likely that such shifts in social identity (i.e., towards a ‘recovery identity’) during lengthier residential treatment might enable a more successful transition into the community and more sustained outcomes [26]. Finally, we did not separate out controlled alcohol use by some participants post-exit, where residents had chosen to incorporate low alcohol use into their recovery, and typically had no problems with alcohol in the past. This suggests that some of the reported findings regarding alcohol use post-exit were conservative, as not all use was ‘problematic’.

### 4.2. Future Directions

The findings of this study have some important clinical implications that warrant further investigation. First, despite severe and complex presentations, the majority of TC residents in this study reported marked reductions and/or abstinence in alcohol or drug use, in addition to improvements across a range of psychosocial outcomes. These changes were sustained at 9 months post-TC exit, suggesting significant lifestyle improvements. The large positive outcomes shown in this study reinforce the value of financial investment in providing long-term residential treatment, and the steep linear ‘dose–response’ association suggests that additional funding to support extended length of treatment would come with both economical and personal benefits, given the high relapse rates for this complex population. However, even longer follow-up periods would enable a greater understanding of the degree to which these outcomes are sustained.

Second, larger cohort studies could assist in being able to examine moderators and mediators of treatment effectiveness, allowing refinements and improvements to TC program content. Finally, the LOS findings were of interest in the context of the development of ever shorter brief interventions for alcohol and drug treatment. Much work is still needed to understand the optimal treatment length for a particular individual by adopting personalised medicine approaches for outcome studies. However, this study suggests that for treatment-seeking individuals with complex presentations, the widespread three-month treatment benchmark [9] may be inadequate. This topic requires careful consideration, further empirical investigation, and discussion with policymakers to ensure we are providing the best possible treatment for this population by reducing unnecessary and costly re-admissions. An area for further work lies in improving employment outcomes for this population post-treatment. Given that employment plays a protective role when improving treatment outcomes, further emphasis on this issue during treatment is warranted.

## 5. Conclusions

Overall, the findings show that residential TC treatment of generally severe and complex presentations was associated with substantial and sustained improvements in consumption (across a range of substances), including impressive levels of drug and alcohol abstinence, reductions in dependence, and improvements in several psychosocial factors at 9 months follow up. Of note, these results suggest that extending the length of stay in TC treatment may reap substantial rewards in the long term.

## Figures and Tables

**Table 1:** Baseline demographics and primary substance use (*n* = 166).

Variable	% (*n*)
Age, Mean (SD)	33.3 (7.70)
Women	35.5 (59)
Single/Separated	76.5 (127)
Born in Australia	83.7 (139)
Highest level of education	
Up to High School	70.4 (117)
Certificate/Trade	22.3 (37)
Tertiary	7.2 (12)
Unemployed	51.8 (86)
Government Benefit recipient	97.0 (161)
Place of Residence	
Owned house	9.0 (15)
Rental/Social Housing	73.4 (122)
Homeless/No fixed address	6.6 (11)
Primary Drug of Choice	
Cannabis	18.7 (31)
Amphetamines	25.9 (43)
Cocaine	0.6 (1)
Sedatives/Tranquilisers	4.8 (8)
Heroin	31.9 (53)
Other opioids/analgesics	4.80 (8)
Alcohol	18.1 (30)
SDS baseline score, Mean (SD) (whole sample)	9.0 (5.3)
SADQ baseline score, Mean (SD) (subsample ^a^)	34.9 (14.4)
Polydrug user ^b^	97.6 (162)
Days in TC at exit, Median (IQR)	206.5 (104.7–370.0)

Note: Unless otherwise specified, results shown are percent (number); SD = Standard Deviation; SDS = Severity of Dependence Scale (Drug dependence); SADQ = Severity of Alcohol Dependence Questionnaire (Alcohol Dependence); ^a^ Subsample identifying as having primary alcohol issue or comorbid alcohol and drug dependence (*n* = 30); ^b^ Alcohol counted towards polydrug status only if respondent reported alcohol as their primary substance; TC = Therapeutic Community; IQR = Interquartile range.

**Table 2:** Drug and alcohol outcomes and clinically significant change.

Variable	Baseline *n* = 166	3 Months Follow-Up *n* = 157	9 Months Follow-Up *n* = 150	Effect Size at 9 Months (Cohen’s *d*)
Drug consumption				
Number drug episodes over 90 days, Mean (SD)	103.1 (85.2)	24.2 (40.5) ***	24.7 (44.5) ***	−0.79
Abstinent from drug use over past 90 days, % (*n*)	12.0 (20) ^b^	49.4% (82) ***	57.1% (95) ***	1.54
Proportion sample over 50% reduction in drug frequency, % (*n*) ^a^	N/A	77.5% (110)	81.7% (116)	N/A
Alcohol Consumption				
Number drinking days over 90 days, Mean (SD)	46.4 (35.3)	18.5 (25.2) ***	19.6 (24.7) ***	−0.47
Number Standard Drinks/drinking day, Mean (SD)	13.6 (11.6)	7.0 (8.9) ***	6.4 (8.2) ***	−0.42
Abstinent from alcohol use over past 90 days, % (*n*)	10.2 (17)	31.9% (53) ***	31.9% (53) ***	0.86
Proportion sample over 50% reduction in alcohol frequency, % (*n*) ^a^	N/A	63.1% (94)	63.8% (95)	N/A
Dependence				
Substance Dependence Scale, Mean (SD)	9.0 (5.3)	4.0 (5.1) ***	3.2 (4.8) ***	−0.56
Severity of Alcohol Dependence Questionnaire, Mean (SD)	19.4 (16.8)	7.5 (9.8) ***	7.3 (9.5) ***	−0.53

Note: *** *p* < 0.001, False Discovery Rate adjusted for multiple comparisons. 3 months follow-up; 9 months follow-up; Significant change in mean consumption at 3 months and 9 months follow-up compared with baseline, using Generalized Linear Models (GLM). See Table S1 for regression tables and model parameters. ^a^ Participants who reported no drugs (*n* = 20) or no alcohol (*n* = 17) at baseline due to prison or other TC admissions were excluded from this analysis. ^b^ Drug use includes participants with a primary alcohol dependence, some reporting drug use at baseline and some not. Note, while some participants were abstinent for drugs or alcohol at baseline, none were abstinent for both. In Australia, one Standard Drink contains 10 g of alcohol (www.alcohol.gov.au).

**Table 3:** Baseline (*n* = 166), 3 months (*n* = 157) and 9 months (*n* = 150) follow-up biopsychosocial outcomes.

Variable	Baseline M (SD)	3 Months Follow-Up M (SD)	9 Months Follow-Up M (SD)	Effect Size at 9 Months (*d*)
Wellbeing ^b,^^	25.18 (18.09)	53.28 (23.45) ***	58.86 (24.70) ***	0.47
Social Function ^a,#^	19.99 (7.73)	14.98 (7.77) ***	13.22 (7.18) ***	−0.23
Psychiatric Status ^c,#^	0.57 (0.22)	0.38 (0.24) ***	0.32 (0.24) ***	−0.32
Employment Status ^c,#^	0.68 (0.29)	0.59 (0.29) **	0.55 (0.31) ***	−0.19
Medical Status ^c,#^	0.18 (0.30)	0.14 (0.27)	0.17 (0.30)	−0.03

Note: ** *p* < 0.01, *** *p* < 0.001, Significance at 3 months and 9 months follow-up compared with baseline, using Generalized Linear Models. ^a^ Social Function subscale of the Opiate Treatment Index (OTI); ^b^ Personal Wellbeing Index (PWI); ^c^ Subscale of the Addiction Severity Index (ASI); ^#^ Higher score on scale indicative of greater problems; ^^^ Higher score on scale indicative of lesser problems. See Table S2 for regression tables and GLM parameters.

## Supplementary Materials

The following are available online at https://www.mdpi.com/2077-0383/9/1/118/s1, Table S1: Generalized linear model analyses for drug and alcohol consumption as a function of time point (*n* = 166), Table S2. Generalized linear model analyses for biopsychosocial outcomes as a function of time point (*n* = 166).
