# Supplementary material for: Longitudinal Substance Use and Biopsychosocial Outcomes Following Therapeutic Community Treatment for Substance Dependence
Source: J Clin Med. 2020 Jan 1;9(1):118. doi: 10.3390/jcm9010118 (PMC7020066; doi:10.3390/jcm9010118)
Supplement: Supplementary file 1 [file jcm-09-00118-s001.pdf]

Table S1. Generalized linear model analyses for drug and alcohol consumption as a function of time point (n = 166).

**Drug episodes** [a]	**B (SE B)**	***t***	**OR**	**95% CI**
Baseline [c]			1.00	
3-month follow up	−1.45 (0.13)	−11.07	0.24 ***	0.18–0.30
9-month follow up	−1.43 (0.14)	−9.92	0.24 ***	0.18–0.32
**Alcohol Frequency (number of drinking days)** [a]	**B (SE B)**	***t***	**OR**	**95%CI of OR**
Baseline [c]			1.00	
3-month follow up	−0.91 (0.11)	−8.03	0.40 ***	0.32–0.50
9-month follow up	−0.85 (0.11)	−8.05	0.43 ***	0.35–0.52
**Alcohol Quantity (mean drinks per drinking day)** [b]	**B (SE B)**	**Wald Chi Sq**	**OR**	**95%CI of OR**
Baseline [c]			1.00	
3-month follow up	−0.67 (0.12)	31.82	0.51 ***	0.40–0.66
9-month follow up	−0.75 (0.12)	39.89	0.47 ***	0.37–0.60
Cannabis episodes [a]	**B (SE B)**	***t***	**OR**	**95% CI**
Baseline [c]			1.00	
3-month follow up	−1.14 (0.16)	−6.96	0.32 ***	0.23–0.44
9-month follow up	−1.51 (0.21)	−7.21	0.22 ***	0.15–0.33
**Heroin episodes** [a]	**B (SE B)**	***t***	**OR**	**95% CI**
Baseline [c]			1.00	
3-month follow up	−1.18 (0.18)	−6.60	0.31 ***	0.22–0.44
9-month follow up	−1.16 (0.20)	−5.74	0.32 ***	0.21–0.47
**Amphetamine episodes** [a]	**B (SE B)**	***t***	**OR**	**95% CI**
Baseline [c]			1.00	
3-month follow up	−1.59 (0.25)	−6.48	0.21 ***	0.13–0.33
9-month follow up	−1.42 (0.25)	−5.73	0.24 ***	0.15–0.4
Primary Drug episodes [a]	**B (SE B)**	***t***	**OR**	**95% CI**
Baseline [c]			1.00	
3-month follow up	−0.99 (0.09)	−11.16	0.37 ***	0.31–0.44
9-month follow up	−0.97 (0.09)	−11.21	0.38 ***	0.32–0.45
**Drug Dependence (SDS)** [b]	**B (SE B)**	**Wald Chi Sq**	**OR**	**95%CI of OR**
Baseline [c]			1.00	
3-month follow up	−0.80 (0.11)	53.94	0.45 ***	0.33–0.62
9-month follow up	−1.03 (0.13)	67.32	0.36 ***	0.26–0.49
**Alcohol Dependence (SADQ)** [b]	**B (SE B)**	**Wald Chi Sq**	**OR**	**95%CI of OR**
Baseline [c]			1.00	
3-month follow up	−0.95 (0.12)	61.20	0.39 ***	0.30–0.49
9-month follow up	−0.98 (0.12)	65.40	0.38 ***	0.29–0.48

*** $p < 0.001$. Note: OR = Odds Ratio; 95% CI = 95% Confidence Interval for odds ratio; [a] Analyses Negative binomial Generalized Linear Models with log link. [b] Analyses Tweedie (1.5) Generalized Linear Models with log link. [c] Reference category.

Table S2. Generalized linear model analyses for biopsychosocial outcomes as a function of time point (n = 166).

**Social Function** [a] [#]	**B (SE B)**	**Wald Chi Sq**	**OR**	**95%CI of OR**
Baseline [c]			1.00	
3-month follow up	−0.29 (0.05)	33.20	0.75 ***	0.67–0.84
9-month follow up	−0.41 (0.05)	64.39	0.66 ***	0.59–0.74
**Psychiatric Status** [b] [#]	**B (SE B)**	**Wald Chi Sq**	**OR**	**95%CI of OR**
Baseline [c]			1.00	
3-month follow up	−0.42 (0.06)	50.82	0.66 ***	0.56–0.78
9-month follow up	−0.59 (0.07)	78.61	0.56 ***	0.47–0.66
**Employment Status** [b] [#]	**B (SE B)**	**Wald Chi Sq**	**OR**	**95%CI of OR**
Baseline [c]			1.00	
3-month follow up	−0.15 (0.05)	8.90	0.86 **	0.76–0.97
9-month follow up	−0.22 (0.05)	16.20	0.80 ***	0.71–0.90
**Medical Status** [b] [#]	**B (SE B)**	**Wald Chi Sq**	**OR**	**95%CI of OR**
Baseline [c]			1.00	
3-month follow up	−0.22 (0.20)	1.20	0.81	0.48–1.35
9-month follow up	−0.06 (0.19)	0.09	0.94	0.57–1.57
**Wellbeing** [c,^]	**B (SE B)**	**Wald Chi Sq**	**OR**	**95%CI of OR**
Baseline [c]			1.00	
3-month follow up	0.75 (0.07)	132.18	2.12 ***	1.85–2.42
9-month follow up	0.85 (0.06)	174.02	2.34 ***	2.05–2.67

** $p < 0.01$. *** $p < 0.001$. Note: OR = Odds Ratio; 95% CI = 95% Confidence Interval for odds ratio; All analyses Tweedie (1.5) Generalized Linear Models with log link. [a] Subscale of the Opiate Treatment Index (OTI); [b] Subscale of the Addiction Severity Index (ASI); [c] Personal Wellbeing Index (PWI); [c] Reference category. [#] Higher score on scale indicative of greater problems; [^] Higher score on scale indicative of fewer problems.
